# Southern Plant Diagnostic Network Invasive Arthropod Workshop: May 7–9, 2007, Clemson, South Carolina, USA

**DOI:** 10.1673/031.009.6101

**Published:** 2009-08-21

**Authors:** Amanda C. Hodges, John C. Morse, Organizers

**Affiliations:** ^1^ University of Florida/IFAS, Entomology & Nematology Department, Southern Plant Diagnostic Network, Gainesville, Florida.; ^2^ Clemson University, Clemson University Arthropod Collection, Department of Entomology, Soils, and Plant Sciences, Clemson, South Carolina.

## Southern Plant Diagnostic Network Entomology Workshop Planning Committee

- Steve Bambara, North Carolina State University
- Carlos Bográn, Texas A&M University
- Eric Day, Virginia Tech
- Keith Douce, University of Georgia
- Frank Hale, University of Tennessee
- Blake Layton, Mississippi State University
- Catharine Mannion, University of Florida
- Blake Newton, University of Kentucky
- Dale Pollet, Louisiana State University
- Charles Ray, Auburn University

## Meeting Website

Oral presentations and image galleries of species discussed are located at: http://www.ipmimages.org/spdn/invasive/

