# Supplementary material for: Southern Plant Diagnostic Network Invasive Arthropod Workshop: May 7–9, 2007, Clemson, South Carolina, USA
Source: J Insect Sci. 2009 Aug 21;9:61. doi: 10.1673/031.009.6101 (PMC3142104; doi:10.1673/031.009.6101)
Supplement: Related texts

# Research article: Current Status of the Distribution of the South American Rice Miner, *Hydrellia wirthi* Korytkowski in Rice in the United States

**Published:** 2009-08

**Authors:** Boris A. Castro, M. O. Way, Wayne N. Mathis, Tadeus Zatwarnicki

**Affiliations:** ^1^ Texas A&M University, Department of Entomology, Agricultural Research and Extension Center, Weslaco, Texas, USA; ^2^ Texas A&M University, Department of Entomology, Agricultural Research and Extension Center, Beaumont, Texas, USA; ^3^ Smithsonian Institution, Department of Entomology, Washington, DC, USA; ^4^ University of Opole, Department of Biosystematics, Poland

The South American rice miner (SARM), *Hydrellia wirthi* Korytkowski, is a new invasive insect pest of rice in the United States. The species was first described from collections in rice fields from Peru and Colombia. It was reported for the first time in the United States from rice fields in Louisiana in 2004. The species was then reported in different rice areas of Louisiana and Texas. The SARM is a shore fly (Diptera: Ephydridae). The only shore-fly species previously known to infest commercial rice in the US was the smaller rice leafminer, *Hydrellia griseola* (Fallén). A field survey for the SARM was conducted in the most important rice producing areas of Louisiana and Texas. The objective of the survey was to determine the distribution of this new invasive species and to assess the severity of infestations in rice fields. Efforts were focused to inspect rice fields from one to six weeks of emergence. Commercial rice fields were scouted using a standard 15-inch sweep net. Ten sweep passes at five different locations were performed in each field. Fly adults (dipterans) were collected from nets using a mouth aspirator. Suspicious dipterans were preserved in 70% ethyl alcohol and forwarded to the USDA-ARS-Systematic Entomology Laboratory (SEL) identifier for official confirmation to species level. In addition, suspected damaged plants were taken to a laboratory and fly larvae reared until adult emergence. Fly adults and associated parasitoids emerged in the laboratory were shipped in vials containing 70% ethyl alcohol. A total of 15 different Louisiana parishes were surveyed which included 1223 hectares (3023 acres) of rice. The following parishes were surveyed in 2005: Vermilion, 260 hectares (642 acres); Acadia, 191 hectares (471 acres); Lafayette, 12 hectares (30 acres); St. Landry, 85 hectares (210 acres); Jefferson Davis, 168 hectares (414 acres), Allen, 103 hectares (254 acres); Calcasieu, 45 hectares (110 acres); St. Martin, 81 hectares (200 acres); Cameron, 81 hectares (200 acres); Evangeline, 12 hectares (30 acres); Avoyelles, 36 hectares (90 acres); Concordia, 133 hectares (328 acres); Tensas, 3 hectares (7 acres); Franklin, 2 hectares (5 acres); East Carroll, 13 hectares (32 acres). Forty five vials containing suspicious fly samples (including 5 vials with emerged parasitoids) were sent to the SEL identifier for identification to species level. The field survey revealed that the SARM is widely distributed in all the important rice producing areas of Louisiana. Higher infestations, i.e. those causing significant yield losses, were observed in coastal parishes including Cameron, Jefferson Davis, Acadia and Vermilion parishes. The insect was found at very low levels in other rice areas of southwest, central and northeastern Louisiana including Calcasieu, Allen, St. Martin, Concordia and Tensas parishes. The distribution of the species in Texas counties includes Calhoun, Colorado, Jackson, Jefferson, Matagorda, and Wharton counties.

# Research article: Heteroptera of Concern to Southeastern U.S.

**Published:** 2009-08

**Authors:** Julieta Brambila

**Affiliations:** USDA APHIS PPQ, Eastern Region, Entomology, Domestic Identifier, P.O. Box 147100, Gainesville, Florida, USA

Twenty-eight species of Heteroptera in five families are discussed and considered as potential or actual pests of economic concern in the United States. The five families are Coreidae, Lygaeidae, Pentatomidae, Scutelleridae, and Tessaratomidae. Three leaf-footed bugs of economic interest within the family Coreidae are *Leptoglossus australis* (F.), *L. occidentalis* (Heidemann), and *L. zonatus* (Dallas). *Leptoglossus australis* is the only species of *Leptoglossus* that does not occur in the Western Hemisphere. The other two bugs are native to Mexico and the western US. *Leptoglossus occidentalis* has been detected in the northeast and has been steadily moving south. The second species, *L. zonatus,* has already been found in Florida and other southeastern states. The family Lygaeidae, known as seed bugs, contains several species of concern to agriculture in the southeast. *Dieuches armatipes* (Walker), a pest of peanuts in Africa, has been in Florida for several years. *Oxycarenus hyalinipennis* Costa, a cotton pest, is known from every continent except North America. It has recently been detected in the Bahamas. Other species of concern include *Blissus antillus* Leonard from the Caribbean, *Dimorphopterus gibbus* (F.) from Africa, Asia and Australia, *Nysius huttoni* Buchanan-White from New Zealand, *Nysius vinitor* Bergroth from Australia, and *Spilostethus pandurus* (Scopoli) from Africa, Asia and Europe. All of these species have shown potential to expand their ranges in recent years. Pentatomids, or stink bugs, that are already in the US and may spread in the southeast are *Euschistus quadrator* Rolston from Mexico and Texas now in Florida and Louisiana; the Asian species *Halyomorpha halys* Stål now in Pennsylvania and New Jersey; and *Oebalus ypsilongriseus* (De Geer) from South and Central America now in Texas and Florida. A serious rice pest that has moved close to the US is *Tibraca limbativentris* Stål, the stem rice stink bug, native to South America; it has been found in the Caribbean. Other exotic species of concern include *Dichelops melacanthus* (Dallas), *D. furcatus* (F.), *Edessa meditabunda* (F.), *Euschistus heros* (F.), *Loxa deducta* Walker and *Oebalus poecilus* (Dallas), all from South America; *Aelia acuminata* L. from Europe and Asia; *Aelia rostrata* Boheman from the Middle East; *Biprorulus bibax* Breddin from Australia; and *Scotinophora lurida* (Burmeister) from Asia. *Eurygaster integnceps* Puton, a shield bug (family Scutelleridae), is considered to be among the world's worst agricultural pests. It feeds on cereal crops, including wheat, throughout its native range in Asia and the Middle East. Although these crops are not abundant in the southeast, early detection of this pest would be critical. *Musgraveia sulciventris* Stål, known in Australia as the Bronze Orange Bug, is a large tessaratomid that damages citrus trees.

# Research article: Exotic Arthropods of Concern to the Southern U.S.

**Published:** 2009-08

**Authors:** Joseph Cavey, Michael D. Petrillo

**Affiliations:** United States Department of Agriculture, Animal Plant Health Inspection Service, Plant Protection and Quarantine, Riverdale, Maryland, USA

Each year, hundreds of exotic insect species approach the United States through international trade and travel. As indicated by pest interceptions made by federal and state inspectors at ports of entry, nurseries and other locations, these potential invaders reflect nearly the entire spectrum of plant pest taxa including obscure species and well documented pests. Among pests of especially high concern, we discuss two in this presentation: *Helicoverpa armigera* (Hubner) and *Anoplophora chinensis* (Forster). Respectively, these species represent exotic pests which arrive with imported consumption products (especially cut flowers) and propagative material (especially bonsai plants). Several other insects which arrive as hitch hikers with non-host commodities are highlighted and their pathways discussed. Criteria used to designate high priority pests are noted and illustrated for each insect; relevant pest interception data are provided and evaluated; and, mitigation measures specific to each of these pests and their pathways are described.

# Research article: Pathways of Introduction and Pest Risk Assessment Issues for Exotic Pests of Regulatory Significance

**Published:** 2009-08

**Authors:** Joseph Cavey

**Affiliations:** United States Department of Agriculture, Animal Plant Health Inspection Service, Plant Protection and Quarantine, Riverdale, Maryland, USA

Increases in the volume and efficiency of international trade and travel have significantly increased the risk and incidence of unintentionally introducing invasive plant pests to the United States. The exact means by which any given adventive species has entered the U.S. is rarely determined. However, patterns of pest entry become evident upon examining pest interception records resulting from inspections of imported commodities from commerce and travelers. Pathways for pest introduction, particularly as defined as a commodity from an origin, present varied degrees of pest risk and require different levels of effort to effectively exclude pests. In this presentation, I review summaries of APHIS, PPQ pest interception data from 1984–2000 reported by McCullough *et al*. (2006) to characterize major pest pathways. Additionally, I briefly discuss certain high risk pest pathways, provide insight on the strengths and limitations of pest interception data for evaluating pathway risk, and outline federal interagency efforts to assess relative pathway risk.

# Research article: Exotic Pest Communications: A Regulatory Perspective

**Published:** 2009-08

**Authors:** Gary L. Clement

**Affiliations:** United States Department of Agriculture, Animal Plant Health Inspection Service, Plant Protection and Quarantine (USDA, APHIS, PPQ), Raleigh, North Carolina, USA

USDA, APHIS, PPQ, safeguards agriculture and natural resources from the risks associated with the entry, establishment, or spread of animal and plant pests and noxious weeds. To carry out this mission, communication channels must be established with stakeholders to deal with exotic pest detections in a timely manner from diagnostic and regulatory perspectives.

PPQ, has traditionally worked closely with the State Departments of Agriculture, in which designated State Plant Regulatory Officials (SPRO), work hand in hand regarding newly detected exotic plant pests within their respective state. From a diagnostic standpoint, PPQ, has its own domestic and port-of-entry identifier specialists that include entomologists, plant pathologists, botanists and mollusk experts. With the continual need for identification services, PPQ, has broadened its diagnostic services reach to university and private laboratories in order to provide timely response to exotic plant pest diagnoses. With the development of the National Plant Diagnostic Network, this additional exotic pest identification resource has increased the ability to identify exotic plant pests of concern to PPQ, and it's stakeholders on a broader scale. After proper identification channels have been followed, final exotic plant pest identification confirmation must be completed by the USDA, ARS Beltsville, MD Laboratory. Regulatory action(s) will be cooperatively determined with the SPRO in the affected state or states in partnership with PPQ. Such actions, based on a through review of the risk associated with a particular exotic plant pest, could involve the establishment of a quarantine area, treatments and/or regulatory actions affecting the movement of infested or infected host material from the established area. The key to successfully addressing new exotic plant pest introductions is open communications between PPQ and cooperators. There is a vital need for such communication to be timely so that needed actions can be implemented in an effective manner.

# Research article: New North American Record of *Megalurothrips mucunae* (Thysanoptera: Terebrantia: Thripidae) in the Southeastern United States

**Published:** 2009-08

**Authors:** Stan Diffie, Joe Funderburk

**Affiliations:** ^1^ University of Georgia, Department of Entomology, Tifton, Georgia, USA; ^2^ University of Florida/IFAS, Northwest Florida REC, Quincy, Florida, USA

The genus *Megalurothrips* is mainly an oriental group with only one of the 12 known species occurring outside the region. None of the species are native to or known to occur in the Western Hemisphere. Routine sampling of weeds in Tift County, Georgia, in April 2005 revealed the presence of *Megalurothrips mucunae*, a new record for this species in North America. Though not detected in other samples in 2005, subsequent collections at multiple locations in Tift County in 2006 confirmed the establishment of this species in the area. The numbers of individuals collected per sample were low on each of the five 2006 sample dates during the spring and fall. The thrips were collected from pine, wild radish, vetch, and snap beans. In September 2006, *M. mucunae* was collected in Quincy, Florida. This collection was made on crepe myrtle, and several hundred individuals were observed on a single date. In another unrelated study in the Smokey Mountain National Park, Tennessee, *M. mucunae* was collected on 3 dates in July 2005, but the specimens were not identified until August 2006. Two color morphs, a light form and a dark form, have been recorded from Georgia and Florida. This species is not recorded in literature as a pest species. However, *Megalurothrips sjostedti* and *M. usitatus* are important pests of legumes in Africa and Asia, respectively. Samples of snap bean flowers in Georgia did reveal the presence of *M. mucunae*. This species should be monitored to detect any damage to beans or other legumes.

# Research article: Thrips

**Published:** 2009-08

**Authors:** Joe Funderburk, Stan Diffie

**Affiliations:** ^1^ University of Florida/IFAS, Northwest Florida REC, Quincy, Florida, USA; ^2^ University of Georgia, Department of Entomology, Tifton, Georgia, USA

The about 5500 recognized species of Thysanoptera are classified into about 750 genera and 9 families. Thrips occupy widely disparate niches with a diversity of life-styles. Thrips are small, opportunistic insects with cryptic habits and can be stealthy insect invaders. The movement of plant species in international trade for over two decades has resulted in the emergence of numerous thrips species as major invasive pests causing extensive crop damage, vectoring of viral diseases, and destabilizing of integrated pest management systems. Over 20 species of thrips are now cosmopolitan. *Frankliniella occidentalis* invaded the southern USA in the 1980's. This is the key vector of *Tomato spotted wilt virus,* the key disease of many ornamental, vegetable, and agronomic crops in the region. *Thrips palmi* is a highly polyphagous pest species that became established in southern Florida in the 1990's. *Scirtothrips dorsalis* is emerging in many parts of the world. It is established in the landscape in Florida where it has already become a major pest of numerous ornamental and vegetable crops. A *Megalurothrips* species recently was established in the southern USA. Numerous other species are invasive in the geographic region, and many species of thrips will emerge as quarantine practices change and the pace of international commerce increases. Early recognition is a challenge. Few specialists are trained in rapid and accurate identification of invasive pest species of thrips. An overview of the morphological characters for identifying thrips to family, genus, and species will be presented that focuses on the important invasive thrips species. Currently, an interactive identification system and information system that provides a fully illustrated and user-friendly means of recognizing economically important thrips is available. Similar identification and information systems are being developed specifically for the southern USA.

# Research article: Ornamental and Turf Pests: Exotic and/or Limited-Distribution Pests of Concern to the Upper South

**Published:** 2009-08

**Authors:** Daniel Gilrein

**Affiliations:** Cornell Cooperative Extension of Suffolk County, Long Island Horticultural Research and Education Center, Riverhead, New York, USA

Movement from recent exotic, pest problems that have established in the Northeastern US is a concern for states in the South. Pest examples discussed included: oriental beetle, European woodwasp, viburnum leaf beetle, Japanense cedar longhorned beetle, common pine shoot beetle, brown marmorated stinkbug, European crane flies, lily leaf beetle, and swede midge. Oriental beetle (*Exomala orientalis*) is a pest of both turfgrass (it is the dominant turf white grub species on Long Island, New York) as well as nursery stock: the grub stage feeds on and damages roots, sometimes killing host plants or areas of turf. Both field- and container-grown nursery stock can be infested. In New York, common woody hosts include rhododendron, azalea, andromeda, junipers and hemlock, and occasional broadleaf species such as cotoneaster. European woodwasp (*Sirex noctilo*) was found in landscape pines near Fulton, New York in September, 2004 and subsequent surveys have documented its presence in much of central New York and a few counties in northern Pennsylvania. It attacks live pines, unlike other native woodwasps, and is associated with the death of pines in Australian and New Zealand plantations where the insect has been introduced, causing up to 80% mortality. Eastern Massachusetts has been battling an invasion of winter moth (*Operophtera brumata*) for at least 15 years. This insect feeds on a wide range of deciduous hosts, such as crabapple, apple, basswood, ash, maples, oak and some spruces. Repeat defoliation of forest and ornamental plants is a concern, but the larvae also feed on very young buds which may threaten blooms and fruit crops such as apple, cherry and blueberry. Portions of the region have sustained heavy defoliation for several years in a row, including an estimated 34,000 acres in Massachusetts in 2005. Winter moth has now spread to new areas in southern New England. Viburnum leaf beetle (*Pyrrhalta viburni*) has spread eastwards from Canada to New England and south into northern Pennsylvania and northeast Ohio. Both adults and larvae feed on and can seriously defoliate and kill a wide variety of viburnum species, including both introduced ornamentals and natives, although some appear to be tolerant or resistant. Japanese cedar longhorned beetle (*Callidiellum rufipenne*) is originally from Asia, but has clearly become established in parts of the northeastern US. In 1998 it was found infesting arborvitae in a Connecticut nursery and subsequent surveys showed it to be established around southern New England, Long Island, New York and New Jersey. Both live and dead plant material have been found infested. Hosts include plants in the families Taxodiaceae and Cupressaceae. Christmas tree and conifer plantations are on alert for common pine shoot beetle (*Tomicus piniperda*) which has spread west, south and east throughout much of the north central and northeastern US, extending as far south as West Virginia. As a quarantine pest, movement of host material from or through affected areas is subject to restrictions. It breeds in dead or dying pines and occasionally other conifers. The adults tunnel into lateral shoots of host plants, causing twigs to die back or break at the point of entry. The damage can spoil the appearance of trimmed Christmas trees or other conifers and in heavy infestations can reduce tree growth. Scots pine is among the most preferred, but Austrian, eastern white, red and jack pine are also hosts. Brown marmorated stinkbug (*Halyomorpha hays*) may be a greater concern in tree fruit, in soybeans and as a household invader, but it can also damage ornamental plants. The adults and nymphs feed on foliage, causing a whitish blotching which might reduce aesthetic value and marketability. This Asian species was believed to be in the Allentown, Pennsylvania area at least since 1996 and has now been found in parts of southeastern Pennsylvania, New Jersey, Maryland, West Virginia, South Carolina and Oregon. European crane flies (*Tipula paludosa* and *T. oleracea*) are reported in parts of the Pacific Northwest, western Canada and New York. The larvae, known as leatherjackets, damage turfgrass (esp. *T. paludosa*), and are also known as pests in pastures and occasionally in greenhouses and nurseries, feeding on stems and roots of container-grown conifer seedlings. Lily leaf beetle (*Liliocens lilii*) larvae and adults defoliate true lilies and *Fritillaria* species. It has now spread throughout eastern Massachusetts and southern Maine, parts of New York, New Hampshire, Rhode Island and Connecticut. Swede midge (*Contarinia nasturtii*) is a pest of cruciferous (cabbage family) plants, including many crop and weed hosts. Although not yet documented, it may also be a pest of ornamental crucifers [ornamental cabbage and kale, *Matthiola* (stocks), *Arabis* (rockcress), *Draba* spp., *Erysimum* spp (wallflower), *Orychophragmus violaceus,* etc.] It has spread around southern Ontario, into Quebec and into 13 counties of central and western New York. The larvae cause severe distortion and stunting of tip growth on host plants.

# Research article: Establishment of *Diaphorina citri* and citrus greening in Florida - A case study

**Published:** 2009-08

**Authors:** Susan Halbert

**Affiliations:** Florida Department of Agriculture and Consumer Services, Division of Plant Industry, Gainesville, Florida USA

*Diaphonna citri* Kuwayama, one of the vectors of the pathogens that cause citrus greening disease (officially huanglongbing), was discovered in Florida in June, 1998 during a routine survey in east-central Palm Beach County. After its range extended to include south Miami-Dade County, it traveled throughout the state on orange jasmine and citrus plants for sale in the discount garden center trade. After the pest became widespread in Florida, it was no longer regulated for intrastate movement. In early 1999, there was a report that citrus greening had been found in Florida. This report prompted extensive survey and testing by laboratories in Florida and around the world. No citrus greening was found in Florida at that time. In particular, there was no sign of citrus greening in east central Palm Beach County where the psyllids became established. Thus, we suspect that the introduction of *D. citri* and of citrus greening probably were independent events. Both the vector and the pathogens likely arrived with illegally imported plant material. Citrus greening was found established in Florida in August 2005 during a targeted survey of Asian farms in south Miami-Dade County. Delimiting surveys were done in southeast Florida (fall, 2005) and southwest Florida (spring, 2006). Anecdotal evidence and the discovery of many severely declining plants indicated that citrus greening probably had been in Florida for 4–5 years prior to discovery. The extent of the distribution, latency of the disease, and public opinion all worked together to make any attempt at eradication unfeasible. Movement of the disease within the state probably occurred via natural flight, trade in orange jasmine and citrus, movement of potted plants by residents, and movement of unprocessed fruit. Citrus greening is a devastating but insidious disease. Current management options are limited and expensive. Once citrus psyllids become established, it may be nearly impossible to prevent establishment of citrus greening, given global movement of plant materials.

# Research article: Invasive hoppers, psyllids and aphids of potential regulatory concern

**Published:** 2009-08

**Authors:** Susan Halbert

**Affiliations:** Florida Department of Agriculture and Consumer Services, Division of Plant Industry, Gainesville, Florida, USA

Several of the world's worst pests are not known to occur in North America. These include *Nephotettix* spp. (Cicadellidae) and *Nilaparvata lugens* (Delphacidae), which are pests of rice. They are key pests in Asia, where they cause direct damage and transmit plant pathogens. Other exotic hoppers also cause damage to crops found in Florida and elsewhere in the southeastern states. *Pyrilla* spp. (Lophopidae) are pests of sugarcane, rice, and maize. Several exotic psyllids are significant pests in South and Central America. *Trioza* spp. (Psyllidae) that colonize avocado in the Neotropics cause galling and other deformation of the leaves. *Russelliana solanicola* (Psyllidae), a South American potato psyllid, causes extensive direct damage to potato crops and probably transmits plant pathogens. The most serious potential exotic aphid pests are Asian species. *Toxoptera odinae* (Aphididae), the mango aphid, colonizes mango and many ornamental pests. *Ceratovacuna lanigera* (Aphididae), the woolly sugarcane aphid, is a key pest of sugarcane in Asia and the Indian Subcontinent

# Research article: Case Study, Hemlock Woolly Adelgid, *Adelges tsugae* Annand

**Published:** 2009-08

**Authors:** Frank A. Hale

**Affiliations:** University of Tennessee Extension, Entomology and Plant Pathology Department, Nashville, Tennessee, USA

The hemlock woolly adelgid (HWA) is originally from Asia. It was first discovered in the United States in Oregon on western hemlock in the 1920s. It was not found in the east until the 1950s, when it was detected in Virginia. Since then it has spread throughout the east from New England to North Carolina (1995), East Tennessee in 2002, and northeastern Georgia soon after. While this pest had been reported to be expanding its range an average of 15 miles per year in northern states, it is spreading at a faster 20–30 miles per year in Georgia. The hemlock species found in Asia and the western United States appear resistant to HWA. Conversely, the eastern and Carolina hemlock found in the East are highly susceptible. Nearly half the hemlock stands in the eastern forests are infested with this pest, which has caused extensive tree mortality, up to 80 percent, in the Middle Atlantic and southern New England states. The insects attach themselves to the base of the hemlock needles and feed from the new twig growth with piercing-sucking mouthparts. The first symptoms are needle yellowing and needle drop, followed by branch desiccation and a lack of vigor indicated by a thinning crown. Limb die-back may occur within two years of the initial infestation on seedlings and saplings. Heavily infested larger trees usually die within four years, although tree death has been reported to occur in just two years in Georgia. Currently, the hemlock trees of the Smoky Mountains National Park (SMNP) and surrounding region are threatened by this pest. Hemlock is an important tree in the ecosystem with no known replacement. Some of the hemlock trees in the SMNP are over 500 years old. The loss of a significant portion of the hemlock in the southern Appalachian region would have profound ecological ramifications. Chemical control is generally effective when used. Unfortunately, most of the infested trees can not be treated. The use of biological control organisms is another option being used in the SMNP and elsewhere. The short and long-term effectiveness of biological control is still being evaluated.

# Research article: NPDN Response Exercise Scenarios: Communication Processes and Protocols in the Event of an Exotic Pest Detection

**Published:** 2009-08

**Authors:** Carrie L. Harmon, Carla Thomas

**Affiliations:** ^1^ University of Florida/IFAS, Department of Plant Pathology, Southern Plant Diagnostic Network, Gainesville, Florida, USA; ^2^ University of California, Department of Plant Pathology, Western Plant Diagnostic Network, Davis, California, USA

The National Plant Diagnostic Network (NPDN) has developed an Exercise Scenario in order to train relevant participants on how to manage secure communications during the initial discovery and confirmatory diagnosis of a plant pest event. The NPDN Standard Operating Procedure (SOP) for APHIS Pests of Concern was the first of its kind to integrate chain of communication and chain of custody procedures from university extension, diagnostic labs at land grant universities and state departments of agriculture, federal confirming diagnostic labs, and state and federal department of agriculture response personnel. This SOP is a living document and undergoes revisions regularly on the basis of lessons learned during exercises and actual events. The activity surrounding a plant pest event has two principal aspects: (a) rapid detection, diagnosis and notification of the pest occurrence, the primary role of the NPDN; and (b) response and regulatory action, which is the coordinated role of the State Plant Regulatory Official (SPRO) and the APHIS-PPQ State Plant Health Director (SPHD) in each state as mandated by regulatory statutes. Key participants involved in exercises include extension, land grant university personnel, state department of agriculture personnel and federal regulatory agency personnel. Since 2003, 42 exercises have been conducted in 44 states and two US territories. Nine states and one territory have completed two exercises, and one state has completed three. Two states have conducted First Detector Exercises. Goals of NPDN exercises include: a) helping all exercise participants (local, extension, state and federal) understand their roles and responsibilities, and how their efforts coordinate with those of the others while practicing standard operating procedures (SOPs) in a non-critical environment, and b) improving the SOPs by identifying and removing ambiguity in the protocols as well as gaps in the procedures. An exercise is composed of three phases: 1) a pre-exercise training session via conference call for all participants, 2) the exercise scenario where participants use the NPDN exercise SOP, 3) a post-exercise debriefing session via conference call to evaluate the exercise and the NPDN exercise SOP. The Plant Diagnostics Information System is a secure website module for monitoring and documenting exercise activities. The website provides critical contact information for the participants, a copy of the SOP to be exercised, an activity log, goals and objectives of the exercise and the photo sheet file that is used as a “plant sample”. The exercise module has been used for all NPDN exercises conducted to date.

# Research article: Invasive scale insects of the southern United States

**Published:** 2009-08

**Authors:** Greg S. Hodges

**Affiliations:** Florida Department of Agriculture and Consumer Services, Division of Plant Industry, Gainesville, Florida USA

Scale insects have historically been considered major agricultural pests and often cause serious problems when introduced into new areas without natural controls. Scale insect introductions into the United States have led to the development of pre and post introduction strategies for exotic pests. The most famous example of an exotic scale insect affecting U.S. agriculture and the development of biological control is the cottony cushion scale with its introduction in 1868 into California. Likewise, the introduction of San Jose scale into California in 1870 helped in the establishment of the U.S. Plant Quarantine Act. In the recent past, there have been 23 new exotic species of scale insects to have been detected in the continental United States. The most notable introductions include: miscanthus mealybug 1989, vine mealybug 1994, litchi scale 1995, cycad Aulacaspis scale 1996, longan scale 1999, lobate lac scale 1999, pink hibiscus mealybug 2002 and white mango scale 2002. The impact of these exotic scale insects is not completely known for all of the species in the south but the biggest impact can be seen with the cycad Aulacaspis scale and pink hibiscus mealybug. In each of these two cases, the most common hosts were relatively free of any major economic pests. After the introduction of the pests, costs of control and economic losses increased to unacceptable levels. Detection and recognition of new pests is paramount now more than ever with the increase in population movement and increase in the ornamental plant industry. The possibility of new exotics finding their way into the southern United States is high. The Cooperative Agricultural Pest Survey (CAPS) program has maintained scale insects on their survey list. The list has incorporated species from four families; Coccidae, Diaspididae, Margarodidae and Pseudococcidae. One of the mealybugs on the list is the passionvine mealybug which was thought to have been found in California in 2006. This resulted in an a more thorough look at current keys available for the determination of this mealybug.

# Research article: Pre and Post-Introduction Strategies for Pink Hibiscus Mealybug. A Case Study

**Published:** 2009-08

**Authors:** Greg S. Hodges

**Affiliations:** Florida Department of Agriculture and Consumer Services, Division of Plant Industry, Gainesville, Florida USA

The pink hibiscus mealybug (PHM) (*Maconellicoccus hirsutus* (Green)) is considered as a major economic pest for many crops grown throughout its range. It is believed to be native to the Orient and is known from over 300 host plant species. It was first found in the Western Hemisphere in Grenada in 1994. With PHM having such a wide host range and being considered as an economic pest, many scientists from the USDA, FDACS-DPI, land grant Universities and from many of the neighboring Caribbean islands visited the Grenada infestation. After viewing the extent of damage from the infestation, a preintroduction strategy started with educational materials being produced for distribution and survey intensity increased in neighboring Caribbean Islands and Florida. This also included an increase on regulations of plant movement and biological control programs also began on Grenada and on neighboring Islands as the mealybug moved around. In Florida, the pre-introduction strategy included the participation of the USDA, FDACS and the University of Florida. The main focus was on educational materials for growers and the public as well as increased intensity of surveys for PHM. As information was made available to the public, a Continental Record did occur for the papaya mealybug (*Paracoccus marginatus* Williams & Granara de Willink) and the first find in Florida for pink hibiscus mealybug occurred in 2002 by a homeowner. Since the first find in 2002, PHM has steadily spread to much of south and central Florida. The post-introduction strategy is ever evolving. It has continued to focus on educational efforts, surveys but has expanded to include chemical and biological controls as well as regulations of plant movement. In 2004, a grower in Florida accidentally shipped potentially infested plant material to 30 plus states. As a response to this, the National Plant Diagnostic Network, National IPM center and the USDA partnered on a USDA-CSREES critical needs grant. This grant sponsored the production of educational materials to be used in the United States and also a emergency workshop that focused on management strategies and identification for pink hibiscus mealybug. There was also an increased awareness within the Florida ornamentals industry that resulted in additional materials being posted on Dr. Lance Osborne's (UF-IFAS) website (http://mrec.ifas.ufl.edu/LSO/Mealybugs.htm) and an increase in regulatory actions on plant movement within Florida.

# Research article: Quick Identification guide to the Wax Scales (Hemiptera: Coccidae: Ceroplastinae) of the United States with comparisons to two exotic species

**Published:** 2009-08

**Authors:** Greg S. Hodges, Michael L. Williams, Amanda C. Hodges

**Affiliations:** ^1^ Florida Department of Agriculture and Consumer Services, Division of Plant Industry, Gainesville, Florida, USA; ^2^ Auburn University, Department of Entomology and Plant Pathology, Auburn, Alabama, USA; ^3^ University of Florida/IFAS, Entomology & Nematology Department, Southern Plant Diagnostic Network, Gainesville, Florida USA

The wax scales (Hemiptera: Coccidae: Ceroplastinae) are some of the most commonly encountered soft scales on ornamental plants in the southern United States. There are thirteen species of wax scales present in the United States and all but one is listed as occurring in the southern region. Species level identification can be difficult for the wax scales and no one concise key exists that addresses both field and slide mounted characteristics for the species found in the United States. In addition to the thirteen species that are present in the United States, there are two species of *Ceroplastes* that are currently on the USDA-CAPS survey list, they are: *Ceroplastes destructor* Newstead (the soft wax scale) and *Ceroplastes japonicus* Green (Japanese wax scale). Both of which superficially resemble species that occur in the United States. Accurate field identification for these two species is generally not possible. In these cases, slide mounted keys are needed. This presentation presents both tentative field and slide mounted keys for identification of the thirteen species of *Ceroplastes* occurring in the United States.

# Research article: Arthropod Pests New to South Florida: What May Be Headed Your Way

**Published:** 2009-08

**Authors:** Adrian G. B. Hunsberger

**Affiliations:** Miami-Dade County Extension, University of Florida/IFAS, Homestead, Florida, USA

South Florida has several major ports of entry and one of the busiest airports in the country allowing for a great influx of International trade and tourism. Along with imported commodities and people, arthropods are frequent hitchhikers, with an average of one new insect species becoming established a month in south Florida. Many of these become either economic or aesthetic pests. The most concerning of these new insects includes: the lobate lac scale (*Paratachardina lobata* (Chamberlin)), pink hibiscus mealybug (*Maconellicoccus hirsutus* (Green)), Sri Lanka weevil (*Myllocerus undatus* Marshall), chili thrips (*Scirtothrips dorsalis* Hood), and weeping fig thrips (*Gynaikothrips uzeli* Zimmerman). All, except for the weeping fig thrips, have a wide host plant range, which includes many commonly grown ornamentals, and pose a significant threat to agricultural crops. In addition, little is known about biology of most of these pests or their potential geographic range. Although it has not been detected in the United States yet, the red palm mite (Raoiella indica Hirst) is expected to arrive shortly.

# Research article: The Japanese Beetle — A Historic Perspective

**Published:** 2009-08

**Authors:** Michael G. Klein

**Affiliations:** The Ohio State University, Department of Entomology, Wooster, Ohio, USA

The Japanese beetle, *Popillia japonica*, was first found in the U.S. at a Riverton, New Jersey nursery just outside of Philadelphia, Pennsylvania in 1916. It was likely brought in with the soil around Japanese iris bulbs sometime before such items were inspected under the Federal Plant Protection Act of 1912. Attempts were made to eradicate the infestation, but little was known about the beetle since it was relatively rare in its natural range from northern Japan up into Siberia. Cool temperatures, lack of grassland, and effective parasitoids kept populations at low levels there. Reports of the Japanese beetle in Korea and China have proved to be mistaken identities with some of the many *Popillia* species found there. In addition to our infestations, the beetles have become established in three of the Portuguese islands of the Azores, and Ontario and Quebec in Canada. All states touching, and east of the Mississippi River, are now likely infested. In addition, Missouri, Kansas, Nebraska, Arkansas, Texas, Oklahoma, Colorado, New Mexico, and Oregon have various population densities. Although only 0.5 sq. miles in New Jersey were infested in 1916, this increased to 5,700 in three states in 1930, 20,611 in eight states in 1941, and 76,504 in 15 states by 1952. Beetles were first found in Missouri in 1949, but that population remained small and isolated in Saint Louis for nearly 50 years before the beetle moved throughout the state. Quarantine programs helped to slow the beetle's spread, but did not prevent it. Two converging events in the 70s have lead to a jump in infested states since that time. First the use of DDT and chlorinated hydrocarbons, which had been very effective in long term control for quarantine programs against Japanese beetle larvae in the soil environment, was withdrawn from the market. This left more toxic phosphates and carbamates which gave weeks or months of control in the soil rather than years. Also then a majority of the states became infested, and a National Japanese Beetle Quarantine was no longer supported. The National Quarantine was replaced by variety of state imposed quarantines and restrictions on the movement of nursery plants and soil. To achieve uniformity in treatments and shipping requirements, a Harmonization Agreement was established to facilitate shipment of nursery stock to western states. This agreement prevented shipment of nursery stock with soil to California, Washington, and Oregon, but allowed shipments to other states following specific treatments. However, no treatments were available which would remove all Japanese beetle larvae from the nursery stock and the beetle rapidly expanded its range to the west. Eradication programs have removed isolated infestations of the beetle as early as 1944 in Novia Scotia, during the 60s, 70s and 80s from three locations in California, and more recently in Nevada and Oregon. The ability of Japanese beetle lures were an intrigal part of the eradication programs by delineating the size and distribution of the populations. Today, many “greener” pesticides such as neonicotinoid compounds and growth regulators are used for control. These materials have been adopted for larval control by home owners and turf managers, but their value in eradication programs is an open question.

# Research article: The Cooperative Agriculture Pest Survey (CAPS) Program: Approach to Surveying for Exotic Species

**Published:** 2009-08

**Authors:** Brian J. Kopper

**Affiliations:** United States Department of Agriculture, Animal Plant Health and Inspection Services, Plant Protection and Quarantine, Raleigh, North Carolina, USA

The Cooperative Agricultural Pest Survey (CAPS) program is a collective effort by Federal and State agricultural organizations to conduct surveys for exotic pests and biological control agents. CAPS surveys can be thought of as our first-line of defense for detecting exotic pests once the cargo has passed through our borders. As such, it is of key importance that survey efforts target pests that have the potential to cause great economic damage while not known to occur (or of limited distribution in) the United States. To this end, the CAPS program strives to find new pests early (prior to establishment) and respond rapidly to any finds so that they can be eradicated before they become widely established. The key to the CAPS program is its cooperative nature. PPQ, establishes partnerships with state departments of agriculture and universities, which through our common goal of early detection, allows us to pool our resources to survey for exotic pests as efficiently and economically as possible. PPQ and our cooperators use two major tools to help in our survey efforts. First, trap locations are determined based on risk. Data (such as pest interception records, climate, host material, etc.) are used to narrow down the possible pest locations in order to place traps in the areas of highest risk. The other tool is public outreach. An aware public can be a valuable tool for detecting exotic pests and mitigating their spread by knowing who to contact if they find something suspicious.

# Research article: Developing a Pest List and Image System to Support the SPDN

**Published:** 2009-08

**Authors:** Joseph H. LaForest, G. Keith Douce, Chuck Bargeron, Amanda Hodges, Carrie Lapaire Harmon

**Affiliations:** ^1^ University of Georgia, Department of Entomology, the Bugwood Network, Tifton, Georgia, USA; ^2^ University of Florida/IFAS, Entomology & Nematology Department, Southern Plant Diagnostic Network, Gainesville, Florida USA; ^3^ University of Florida/IFAS, Department of Plant Pathology, Southern Plant Diagnostic Network, Gainesville, Florida, USA

The Bugwood Network (www.bugwood.org) has partnered with The Southern Plant Diagnostic Network (SPDN) to develop a comprehensive list of organisms-of-interest to SPDN. This list is being used to solicit images to populate the IPMImages image archive system (www.ipmimages.org) to support SPDN training and educational programs. This project builds upon the successful Bugwood Network image system that provides high resolution, identified and credited images that are available at no-cost for educational uses. The Bugwood image system currently contains more than 54,000 images on 9,000 subjects that have been taken by over 1,100 contributors in 45 countries. Bugwood web sites received 118 million hits during 2006. The Bugwood Network — SPDN partnership has been made possible through a CSREES Southern Region IPM project with objectives to: 1) identify the species of concern to the SPDN, 2) obtain a comprehensive set of images of the listed species including all life stages and depictions of damage and 3) provide an interface to easily access these images as well as others to be used in the SPDN training and education modules. The list includes native and non-native pests as well as biological controls found in the Southern region. It also includes exotic species that they are not currently present, but may pose a significant threat if introduced. The end result will be a useful tool to aid diagnosticians and a reliable source of quality, high-resolution images for anyone creating publications, presentations and other educational materials. The organisms-of-interest list contains 252 insects and 96 pathogens and is available at www.ipmimages.org/spdn. It is categorized by pest status (exotic, native, introduced), commodity and the number of images available for each life stage. Project personnel are soliciting image contributions for each species to include whole-organism images of each life stage, damage symptoms, as well as diagnostic features and characteristics. All images obtained through this project will be incorporated into and made available for SPDN and other educational and training needs through IPMImages.

# Research article: Laurel Wilt: A New Disease Threatening Redbay and Other Plants in the Lauraceae

**Published:** 2009-08

**Authors:** Albert E. Mayfield, Stephen W. Fraedrich, James Johnson, Laurie S. Reid, Jason A. Smith

**Affiliations:** ^1^ Florida Department of Agriculture and Consumer Services, Division of Forestry, Gainesville, Florida, USA; ^2^ Southern Research Station, USDA Forest Service, Athens, Georgia, USA; ^3^ Georgia Forestry Commission, Athens, Georgia, USA; ^4^ South Carolina Forestry Commission, Columbia, South Carolina, USA; ^5^ School of Forest Resources and Conservation, University of Florida, Gainesville, Florida, USA

Laurel wilt is a new vascualar disease of redbay (*Persea borbonia*) and other plant species in the family *Lauraceae.* The disease is caused by a fungus (*Raffaelea* sp.) that is introduced into host trees by a non-native vector, the redbay ambrosia beetle (*Xyleborus glabratus*). The redbay ambrosia beetle was first detected in the U.S. near Savannah, Georgia in 2002. Laurel wilt has caused high levels of redbay mortality in coastal regions South Carolina, Georgia, and Florida and by January 2007 had spread to at least 31 counties. Affected redbays exhibit wilted foliage and dark streaks of discoloration in the sapwood. The disease has also been detected in related species, including sassafras (*Sassafras albidum*), pondspice (*Litsea aestivalis*)*,* avocado (*Persea americana*) and the endangered pondberry (*Lindera melissifolia*) in the field. Current management options for laurel wilt are extremely limited and the distribution of this disease is expected to continue to expand.

# Research article: Weevils (Curculionoidea): Invasive Species New and Old

**Published:** 2009-08

**Authors:** Charles W. O'Brien

**Affiliations:** University of Arizona, Department of Entomology, Tucson, Arizona, USA

The weevils represent one of the most important groups of invasive species, and new pest species arrive frequently in these days of modern transportation. In the past they came by ship and arrived with early seafarers and with the settlers. Many benefit as invaders by their parthenogenetic biology's and tough adult cuticle and long lives. Included in this presentation are some species of great importance and others with the potential to become serious pests, e.g. Myllocerus undatus Marshall; *Listroderes difficilis* Germain; *Sitona lineatus* (L.); *Pseudocneorhinus bifasciatus* Roelofs; *Trachyphloeosoma advena* Zimmerman; *Diaprepes abbreviatus* (L.); *Naupactus cervinus* (Boheman); *Naupactus leucoloma* (Boheman); *Naupactus peregrinus* (Buchanan); *Cyrtepistomus castaneus* (Roelofs); *Calomycterus setarius* Roelofs; *Cylasformicarius* (Fabricius); *Oedophrys hilleri* (Faust); *Polytus mellerborgii* (Boheman).

# Research article: Characters and Techniques for Identifying Lepidoptera of Quarantine Significance to the Southern United States from USDA/APHIS/PPQ CAPS Programs

**Published:** 2009-08

**Authors:** Steven Passoa

**Affiliations:** USDA/APHIS/PPQ, Columbus, Ohio, USA

Identification characters and techniques for Lepidoptera are discussed. Eggs are slide mounted to examine the micropyle and chorion. Preserved larvae of leaf miners should be associated with the pressed mine of the host. Larvae are studied by slide mounting the body and mouthparts. Pupal identification is more accurate if the larval skin and cocoon are not discarded. Submit cast pupal skins dry. If the pupal eye is dark, there is a good chance the genitalia of the pharate adult can be dissected and examined. Immatures are best reared to adult; this is especially true of eggs. Sticky traps should never be sealed in plastic bags, folded one on top of the other, or sealed with heavy duty staples. Instead, fold them in a triangle when viewed in cross section, then close with weak scotch tape. Screen samples and only forward those closely related the target. Traps should be labeled with locality data, a unique code number, and the target pest for the survey. A “mini pest risk assessment”, using criteria I developed as modified by members of the Northeast Exotic Pest Survey Committee in 1992, was applied to Lepidoptera of regulatory concern to the southern United States. The criteria were: tolerance to the southern US climate, host availability, survey methodology, ease of identification, availability of a high hazard site, potential economic impact, and an evaluation of the entry and establishment potential of the pest. Lepidoptera on corn, sorghum, cotton, citrus, sugarcane, soybean and ornamentals were favored. Two categories of pests were noted (threats and CAPS targets). A threat is defined as a species with a negative impact to the environment, trade, or the agroecosystem. A CAPS target should also be practical to identify, be frequently intercepted or have a good pathway to enter the USA, and have a high probability of establishment after arrival. Selected pests meeting these criteria that either are not in the United States, or are of limited distribution, include: *Opogona sacchan* (Tineidae); *Phyllocnistis citrella* (Gracillariidae); *Blastobasis graminea* (Coleophoridae); *Pectinophora scutigera, Platyedra subcinerea* (Gelechiidae); *Acrolepiopsis assectella* (Acrolepiidae); *Prays citri* (Yponomeutidae); *Synanthedon myopaeformis* (Sesiidae); *Pryena sinica* (Zygaenidae); *Darna pallivitta* (Limacodidae); *Thaumatotibia leucotreta, Epiphyas postvittana, Epinotia aporema* (Tortricidae); *Papilio demoleus* (Papilionidae); *Maruca vitrata, Duponcheliafovealis, Diaphania indica, Chilo spp., Eoreuma lofiini* (Crambidae); *Cryptoblabes gnidiella, Cactoblastis cactorum* (Pyralidae); *Lymantria spp.* (Lymantriidae); *Dendrolimus spp.* (Lasiocampidae); *Spodoptera spp., Autographa gamma, Helicoverpa armigera, Copitarsia sp., Chrysodeixis chalcites, Cornutiplusia circumflexa, Mamestra brassicae, Metaponpneumata rogenhoferi, Sesamia spp. Busseola spp*., and *Noctua pronuba* (Noctuidae). Simplified screening keys to superfamilies and characters or references useful for species identification are provided for the most frequently seen life stage of the above taxa. The proboscis, tympanum, chaetosema, genitalia, pilifers, pupal labial palpi and abdominal spines, prespiracular group, larval mandibles and spinneret, abdominal L setae, proleg number and crochet arrangement are the most important characters.

# Research article: Emerald ash borer, Agrilus planipennis, attraction in field trapping experiments employing combinations of visual, olfactory, tactile and canopy position cues

**Published:** 2009-08

**Authors:** Therese M. Poland, Deepa Pureswaran, Peter de Groot, Gary Grant, Deborah G. McCullough

**Affiliations:** ^1^ USDA Forest Service, North Central Research Station, East Lansing, Michigan, USA; ^2^ Michigan State University, Department of Entomology, East Lansing, Michigan, USA; ^3^ Department of Natural Resources, Canadian Forest Service, Sault Ste. Marie, Ontario

Since its discovery in Southeast Michigan in 2002, the emerald ash borer (EAB), *Agrilus planipennis* Fairmaire (Coleoptera: Buprestidae), appears to be living up to expectations and predictions about its potential spread and destruction of ash trees, *Fraxinus* spp., in North America. Currently, the generally infested “core” area includes 21 counties in Southeast Michigan and extends into Ohio and Ontario, Canada. In addition, numerous outlier infestations have been found throughout Michigan's Lower Peninsula, Ohio, Indiana, and Ontario. An outlier in Maryland that originated from infested Michigan nursery stock was first detected in 2003 and is still under eradication. Two isolated infestations were detected in the Chicago area of Illinois in the summer of 2006. As of 1 December 2006, APHIS revised the federal quarantine to include the entire states of Ohio, Indiana, and Illinois, in addition to the Lower Peninsula of Michigan. State quarantines further restrict within state movement of ash. The area infested with EAB now exceeds 40,000 square miles in Michigan, Ohio, Indiana and Ontario and it is estimated that the beetle has killed more than 20 million ash trees in the core infested area of the US. Early detection of emerald ash borer (EAB), *Agrilus planipennis* Fairmaire (Coleoptera: Buprestidae), remains a major challenge for regulatory officials due to the delay in onset of visual symptoms of attack. Currently, regulatory and resource management agencies rely on girdled trap trees for statewide survey and detection programs. However, locating suitable detection trees can be difficult and felling and debarking trees to locate galleries is labor intensive and costly. Development of effective traps and attractants is a high priority for the EAB management program. Previous studies suggest that *A. planipennis* is attracted to the color purple, to blends of host volatiles from ash bark and foliage, and to girdled ash trees. It has also been found that *A. planipennis* prefers to attack trees with rough bark. We evaluated attraction of *A. planipennis* to triangular purple panel traps baited with various combinations of ash volatiles that elicit antennal responses by *A. planipennis* as well as volatiles from ash that have behavioral activity in other bark or wood-boring insects. We also evaluated attraction of *A. planipennis* to traps that incorporated multiple components of attraction. Multicomponent traps included triangular purple panels mounted at 1.5 and 2.5 m on a purple pole. The upper and lower panels were baited with foliar and bark volatiles, respectively. Panels were also coated with a rough “bark” texture. Treatments included binary and ternary combinations of foliar volatiles, bark volatiles, and texture. Overall, we captured more than 4,000 *A. planipennis* on the 160 multi-component traps used in the experiment. Traps baited with both the foliar volatiles and bark volatiles but without texture captured the most *A. planipennis.* Traps baited with the bark volatiles alone and coated with the texture caught significantly fewer *A. planipennis* than traps baited with both bark and foliar volatiles but without texture. Traps coated with texture and baited with either the leaf volatiles alone or the leaf and bark volatiles caught an intermediate number of *A. planipennis.* The combined effect of omitting the leaf volatiles and including texture significantly reduced attraction compared to traps with both the leaf and bark volatiles but no texture. Therefore, it appears that leaf volatiles are an important factor in *A. planipennis* attraction. The texture that was applied to the traps may have interfered with *A. planipennis* capture, due to reduced adhesion of the beetles to the rough surface which was difficult to coat with Pestick. Thus, bark roughness is probably not an important factor in long range attraction of *A. planipennis*, but is more likely involved in post-landing acceptance of oviposition sites. The results will lead to improved trapping and detection methods for *A. planipennis*.

# Research article: Non-native Forest Pests of Concern

**Published:** 2009-08

**Authors:** Bob Rabaglia

**Affiliations:** USDA Forest Service, Forest Health Protection, Arlington, Virginia, USA

Non-native forest insects and pathogens have had a significant impact on North American rural and urban forests. From chestnut blight to emerald ash borer, these pests have affected the ecology and economy of our forest communities. In the past decade there have been an increasing number of new, non-native forest pests of concern. The current status of Sirex woodwasp, emerald ash borer and several ambrosia beetles will be discussed, as well as options available for their management.

# Research article: Mite Threats to the Southeastern USA

**Published:** 2009-08

**Authors:** Cal Welbourn

**Affiliations:** Florida Department of Agriculture and Consumer Services, Division of Plant Industry, Gainesville, Florida, USA

Plants host a wide variety of mites (Acari) including phytophagous groups (Tetranychoidea, Eriophyoidea and Tarsonemidae), predators (Phytoseiidae, Anystidae, Bdellidae, and Cunaxidae), the ubiquitous Tydeidae and other less common groups. North America is under constant threat from exotic mites that can enter on plants, in containers and carried by unsuspecting travelers. Some of the many pest species that could be significant pests if they were to become established in the USA include: *Eutetranychus onentalis* (Klein), *Brevipalpus chilensis* Baker, *Raoiella indica* Hirst, and *Stenotarsonemus spinki* Smiley. The red palm mite, *Raoiella indica* Hirst, is an example of a mite that was not considered to be a serious threat to the USA until it was reported from the southern Caribbean in 2004. Since then red palm mite has become one of the most numerous mites in the Caribbean region. In 2006 it reached Puerto Rico and the Dominican Republic and now threatens the native and ornamental palms in the Southeastern USA. Another example is *Brevipalpus phoenicis* (Geijskes), a common plant pest in many parts of the world. The threat to the southeastern USA is not the presence of this mite, but the fact that it is a vector of Citrus Leprosis. Other phytophagous mites (i.e. *Eutetranychus onentalis* (Klein)) pose significant treats because of their wide host range. Since most phytophagous mites look the same to the unaided eye it would be easy for an exotic mite to go unnoticed until it became a serious problem.

# Research article: Spiders, Sowbugs, and Earthworms as Invaders and their Impact

**Published:** 2009-08

**Authors:** Robert J. Wolff

**Affiliations:** South University, DoctorSpider LLC, and Adjunct Clemson University, Columbia, South Carolina, USA

Spiders (Order Araneae) are generally not considered important as invasives, but recent range expansions of the Brown Widow Spider and Hobo spider are of concern due to their reported toxic bites. An exotic tarantula and a brown spider (*Laxoceles*) have both become established in the U.S. among many other species that are of less concern. Sowbugs (woodlice, Order Isopoda) have not raised alarms, but are actually of concern due to their impact on the organic layers of the soil. Most of the species in the U.S. are exotics, and they have come to dominate the soil fauna in most locations. Exactly what their ecological impact is on the plant and animal communities is unknown, they do speed the breakdown of the detritus. Exotic earthworms (Class Oligochaeta) on the other hand cause tremendous ecological damage to soils and their communities. They are similar in that they now dominate most soil communities and are also found in very high populations. They have even changed soil structure so drastically that the soil belongs to a different order following invasion, and have led to localized extinctions of plant species. Their ecological impact affects all plant and animal species, including increasing wildlife species that feed on earthworms. Support in part comes from the National Park Service R5240050019 [CESU-CU COSW].
